# X-ray Study of Structural Formation and Thermomechanical Properties of Silver-Containing Polymer Nanocomposites

**DOI:** 10.1186/s11671-017-1967-2

**Published:** 2017-03-31

**Authors:** Valeriy Demchenko, Sergii Riabov, Volodymyr Shtompel’

**Affiliations:** Department of Polymers Modification, Institute of Macromolecular Chemistry, The National Academy of Sciences of Ukraine, 48, Kharkivske chaussee, Kyiv, 02160 Ukraine

**Keywords:** Interpolyelectrolyte complexes, Interpolyelectrolyte–metal complexes, Nanocomposite, Structure, Thermomechanical properties

## Abstract

The structural organization and thermomechanical properties of nanocomposites prepared from interpolyelectrolyte–metal complex (IMC) involving anionic polyelectrolyte, pectin and AgNO_3_, and cationic polyelectrolyte, poly(4-vinylpyridine), have been investigated using the methods of wide- and small-angle X-ray scattering and thermomechanical analysis. It is established that chemical reduction of Ag^+^ ions in the IMC by sodium borohydride results in formation of the nanocomposite based on the “pectin–poly(4-vinylpyridine)” interpolyelectrolyte complex (IPEC) and Ag^0^ nanoparticles as well. At the same time, the level of nanocomposites’ structural heterogeneity is substantially enhancing, while effective size of the heterogeneity regions decreases. The nanocomposites IPEC–Ag^0^ prepared are shown much bigger *T*_*g*_ value and enhanced ability for deformation than those for IMC.

## Background

During the last decades, the interest in studying of nanosize particles of different metals and their oxides has constantly grown up [1–4]. Primarily, it is due to their unique characteristics, dramatically differing from their analogs—micro-scale size objects. So, this fact opens a new possibility for diverse applications of nanomaterials, having advanced properties in an industrial area.

Hybrid materials, containing silver nanoparticles, show promising features for the design of catalytic systems, and they are currently used in optoelectronics and nanophotonics [5–10]. In turn, nanocomposite materials [10–12] with silver nanoparticles have found a wide application as effective antibacterial and antiviral preparations. As for the other metal colloids, it should be emphasized that the size and size distributions of silver nanoparticles and their stability are determined by the nature of the stabilizing polymer matrix, the molecular ratio of metal ions and macromolecular units, and the conditions of reduction of silver ions [10–17].

Developing of such materials is not possible without a fundamental researches and studying their structure and physico-chemical and mechanical properties.

The current review of scientific sources revealed that data concerning investigations of a structural organization and physical and mechanical properties of Ag^0^-containing nanocomposites obtained by chemical reduction of Ag^+^ ions in the interpolyelectrolyte–metal complexes are not published yet.

So, the aim of this work is to study the structural organization and thermomechanical properties of nanocomposites involving a natural and synthetic polymers—pectin, poly(4-vinylpyridine), and Ag^0^ nanoparticles—formed from interpolyelectrolyte–metal complexes.

## Methods

### Materials

To obtain the interpolyelectrolyte complexes (IPEC), pectin–poly(4-vinylpyridine) (P4VP); the interpolyelectrolyte–metal complexes (IMC), pectin–Ag^+^–P4VP; and nanocomposites of IPEC–Ag^0^, the following reagents were used: pectin sodium salt (Na-pectin)—obtained by mixing citrus pectin production “Cargill Deutschland GmbH” (Germany), *M* = 3 × 10^4^, with NaOH; hydrochloride poly(4-vinylpyridine) (P4VP-Cl)—received protonation pyridine ring poly(4-vinylpyridine) (Aldrich), *M*_*w*_ = 6 × 10^4^, with HCl; silver (I) nitrate (AgNO_3_) (Aldrich) with *M* = 169.9 and sodium borohydride (NaBH_4_) (Aldrich) with *M* = 37.83.

### Preparation of Polymer Systems

IPEC samples were formed via mixing of 5% aqueous solutions of Na-pectin and P4VP-Cl taken at an equimolar ratio, at *T* = 20 ± 2 °C.


While mixing of anion and cation polyelectrolytes’ (PE) water solutions, one could observe immediate formation of clots as a result of a process of molecular “recognition” and self-assembly of oppositely charged PE macromolecules [18]. These clots, which are IPEC, were formed as thin films on polytetrafluorethylene plates, dried at *T* = 20 ± 2 °C to a constant mass, then washed in distilled water getting reached neutral pH, and then dried again till to a constant mass. The resulting films were 100–500 μm thick.

IMC samples were prepared via immersion of IPEC films into an aqueous solution of AgNO_3_ with a concentration of 0.1 mol/L at *T* = 20 ± 2 °C for 24 h. The colorless IPEC films became dark red.

The absorption capacities of films, *A* (mmol/g), were calculated through the formula [19]

$$ A=\left({c}_{\mathrm{in}}\hbox{--}\ {c}_{\mathrm{eq}}\right) V/ m, $$

where *m* is the mass of the absorbent, *V* is the solution volume, and *c*_in_ and *c*_eq_ are the initial and the equilibrium concentrations of silver ions, respectively. For IMC films, *A =* 2.2 mmol/g.

The chemical reduction of Ag^+^ ions in the IMC was realized with NaBH_4_ (a molar ratio of [BH_4_^–^]:[Cu^2+^] ≥2.0) in an alkaline medium (pH 10.8) in a solvent mixture of water–isopropanol (4:1 vol%) at *T* = 20 ± 2 °C for 3 h (until the release of gaseous bubbles ceased). The concentration of NaBH_4_ in the aqueous alcohol solution was 0.1 mol/L. As a result of the reduction, IMC films changed color from red to a metallic silver one.

### Experimental Methods

The features of the structuring of the IPEC (pectin–P4VP), the IMC (pectin–Ag^+^–P4VP), and nanocomposites of IPEC–Ag^0^ were studied by wide-angle X-ray scattering (WAXS) with a DRON-4-07 diffractometer (scientific-production company “Burevestnik,” Russia), whose X-ray optical scheme was used to “pass” primary-beam radiation through samples.

The heterogeneous structuring of these polymeric systems (at the nanometer level) was studied via small-angle X-ray scattering (SAXS) with a CRM-1 camera (Orel scientific equipment factory, Russia), having a slit collimator of the primary irradiation beam made via the Kratky method. The geometric parameters of the camera satisfied the condition of infinite height of the primary beam [20]. The intensity profiles were normalized to the volume of X-ray scattering and the attenuation factor of the primary beam of the test sample.

All X-ray diffraction studies were performed at *T* = 20 ± 2 °C in Cu*К*_α_ radiation monochromated with a Ni filter.

Thermomechanical studies of polymer systems were conducted using the penetration method in the mode of a uniaxial constant load (*σ* = 0.5 MPa) with a UIP-70M device (central design engineering bureau of the special instrument making of the National Academy of Sciences of Russia). Linear heating of samples was performed at a rate of 2.5 °C/min in the temperature range 0 to +250 °C. Relative penetration (%) was calculated as

$$ \varepsilon =\left(\varDelta l/{l}_0\right)\cdot 100, $$

where ∆*l* is the penetration (μm) at certain temperature and *l*_0_ is the initial thickness of the sample (μm).

## Results and Discussion

**Fig. 1:**
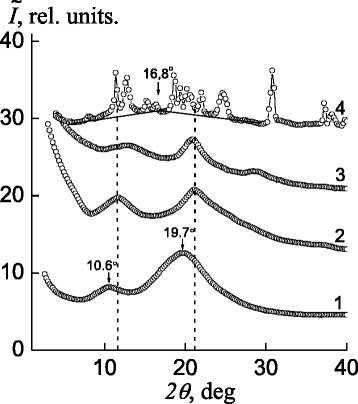
WAXS of *1* P4VP, *2* the IPEC pectin–P4VP, *3* a pectin film, and *4* a pectin powder

Comparing of the WAXS diffractograms of the cationic and anionic polyelectrolytes on whose basis the IPEC was formed (see Fig. 1), it was found that P4VP has only short-range ordering while translated in bulk a fragments of the main macromolecular chains and their lateral branches—pyridine ring (curve 1), and pectin has an amorphous–crystalline structure (curve 4).

The analysis of the atomic structure of macromolecules P4VP showed that low-intensity peak at 2*θ*_max_ ~10.6° describes the near arrangement of basic macromolecular chains, while 2*θ*_max_ ~19.7° characterizes pyridine ring (Fig. 1, curve 1).

Herewith, the average value of *d* period of the short-range ordering of fragments of basic macrochains at their positioning in a space (in a volume of P4VP) according to the Bragg equation is

$$ d=\lambda {\left(2 \sin {\theta}_m\right)}^{-1}, $$

where *λ* is the wavelength of the characteristic X-ray radiation, which is 8.3 Å, whereas Bragg in space at rotation cycles of pyridine—4.5 Å (*λ* = 1.54 Å for Cu*K*_α_ radiation).

On the X-ray profile of pectin, whose sample is a powder (Fig. 1, curve 4), there are many singlet and multiplet diffraction maxima having an imaginary amorphous halo with a vertex at 2*θ*_*m*_ ~16.8° in the background, indicating the amorphous–crystalline structure of this polysaccharide.

The relative crystallinity of pectin, *X*_cr_, was determined via the Mathews method [21]:

$$ {X}_{\mathrm{cr}}={Q}_{\mathrm{cr}}{\left({Q}_{\mathrm{cr}}+{Q}_{\mathrm{am}}\right)}^{\hbox{--} 1}\cdot 100, $$

where *Q*_cr_ is the area of the diffraction maxima, characterizing the crystalline structure of the polymer, and *Q*_cr_ + *Q*_am_ is the total area of the X-ray pattern in the range of scattering angles 2*θ*_1_ *÷* 2*θ*_2_, where the amorphous–crystalline structure of the polymer manifests itself. The calculations showed that *X*_cr_ approximates the value of 65%.

In turn, effective crystallite size *L* of pectin determined via the Scherrer method [22]

$$ L=\mathrm{K}\lambda {\left(\beta \cos {\theta}_m\right)}^{\hbox{--} 1}, $$

where *К* is a constant related to a shape of the crystallites (*K* = 0.9 if their shape is unknown) and *β* is the angular half-width (width at a half-height) of the singlet of a discrete diffraction maximum, showing that the average *L* value is 17.5 nm (for the calculation, singlet diffraction maxima at 2*θ*_*m*_ = 18.7° and 30.8° were applied).

However, the X-ray pattern of the pectin sample in the form of a film prepared from a 5% aqueous solution displays only contours of the groups of diffraction maxima with basic intensities that are present on the X-ray pattern of the pectin powder (Fig. 1, curves 3 and 4). This circumstance indicates a low rate of pectin crystallization, as well as the relaxation character of the structurization processes in the polymers.

IPEC formed from pectin and P4VP is characterized by short-range ordering during translation of fragments of oppositely charged polyelectrolyte macromolecular chains in a space. This fact is confirmed by the appearance of diffuse diffraction maximum with 2*θ*_*m*_ ~21.2° on the X-ray profile of the IPEC sample (see Fig. 1, curve 2). The average value for the period of short-range ordering of macromolecular chains’ fragments of oppositely charged polyelectrolytes in the IPEC (the average Bragg distance between chains of the polyanion and the polycation) is equal to 4.2 Å.

One should pay attention to the following fact: in the IPEC profile obtained for equimolar quantity of pectin and P4VP, the angular location of the secondary (rated by intensity) diffraction maximum is shifted from 10.6° to 11.8° comparing with P4VP (Fig. 1, curves 1 and 2), while the average Bragg distance (*d*) between P4VP’s main macrochains (as part of IPEC) is falling down from 8.3 Å to 7.5 Å.

**Fig. 2:**
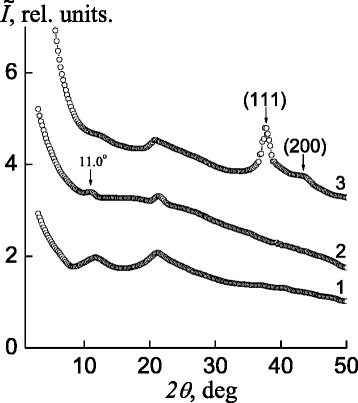
WAXS patterns of *1* the IPEC pectin–P4VP, *2* the IMC pectin–Ag^+^–P4VP, and *3* the IPEC–Ag^0^ nanocomposite

Once the pectin–Ag^+^–P4VP IMC is formed, the diffraction pattern changes. This is confirmed by the appearance of a low-intense diffuse diffraction maximum at 2*θ*_*m*_ ~11.0° (Fig. 2, curve 2) in the presence of a low-intensity amorphous halo, which, unlike that for the IPEC, has an angular position at 2*θ*_*m*_ ~21.4°. This diffraction maximum, according to [1], characterizes the existence of interpolyelectrolyte–metal complexes between the ions (Ag^+^) and ligands. Taking into account the angular position of this diffraction peak on the X-ray diffractogram of the IMC, average Bragg distance *d* between the macromolecular chains of polyelectrolytes coordinated with Ag^+^ ions is found to be 8.0 Å.

Chemical reduction of the Ag^+^ ions in the IMC by sodium borohydride results in the formation of a nanocomposite based on the IPEC and Ag^0^. In the nanocomposite’s profile (Fig. 2, curve 3), one can see the low-intense diffraction maximum at 2*θ*_*m*_ ~11.0°, which is typical of the above interpolyelectrolyte–metal complexes, is absent, unlike two intensity diffraction peaks appeared at 2*θ*_*m*_ ~37.8° and 43.6°, corresponding to the crystallographic plan of the face-centered cubic lattice of silver with (111) and (200) indexes, respectively, thus confirming presence of metallic silver in the polymeric system. Moreover, diffraction maximum intensity at 2*θ*_*m*_ ≈ 20.8^o^, characterizing structure of IPEC “pectin–P4VP,” is enhanced.

Effective size *L* of Ag^0^ nanoparticle crystallites in the IPEC proved to be 4.0 nm (for the calculation, diffraction maxima at 2*θ*_*m*_ = 37.8° and 43.6° were used, curve 3).

The revealed peculiarities and changes in structures while transiting from the IPEC to IMC and IPEC–Ag^0^ nanocomposites form the basis for the further study of their structural heterogeneity.

**Fig. 3:**
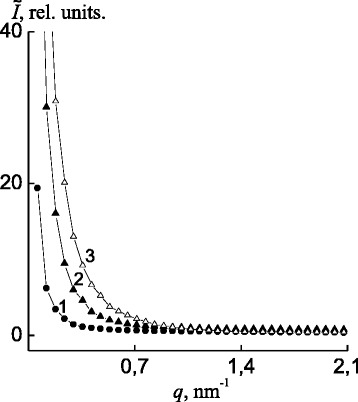
SAXS-intensity profile patterns of *1* the IPEC pectin–P4VP, *2* the IMC pectin–Ag^+^–P4VP, and the *3* IPEC–Ag^0^ nanocomposite

Analyzing the profiles of small-angle X-ray scattering of the polymer systems, presented in [23, 24], as dependences of *Ĩ* on *q* (Fig. 3) and *s* ^3^ *Ĩ* on *s* ^3^, where *Ĩ* is the intensity of scattering without the collimation correction and *q* = (4*π*/*λ*)sin*θ* = 2*πs*, all of these systems have heterogeneous structure, i.e., contrast electron densities Δ*ρ* (Δ*ρ* = *ρ* − <*ρ>*, where *ρ* and *<ρ>* are the local and average values of the electron density, respectively) are present in their volumes. This result means that in the IPEC, IMC, and the nanocomposites based on the IPEC and Ag^0^, there exist at least two types of region’s heterogeneity with different values of local electron density *ρ*.

As one can see, scattering intensity and thus Δ*ρ* value are increasing for these systems in the following rank: IPEC pectin P4VP → IMC pectin–Ag^+^–P4VP → nanocomposite IPEC–Ag^0^ (Fig. 3, curves 1*–*3). However, the absence of the interference peak in the all profiles indicates the stochastic nature of the location of various types of heterogeneity areas in a space.

In order to assess semi-quantitatively the value of the relative level of structural heterogeneity of these polymer systems, their Porod invariants *Q*′ were compared [25]:

$$ Q^{\prime }={\displaystyle \underset{0}{\overset{\infty }{\int }} q\tilde{I}(q)} d q, $$

These values are invariant with respect to the shapes of the heterogeneity regions and are directly related to the rms values of electron density fluctuations (*<*Δ*ρ* ^2^ *>*) in a two-phase system:

$$ Q^{\prime}\propto <\varDelta {\rho}^2>, $$

where *<*Δ*ρ* ^2^ *> = φ*_1_ *φ*_2_(*ρ*_1_ *–ρ*_2_)^2^, where *φ*_1_ and *φ*_2_ are the volume ratios of heterogeneity domains in a two-phase system and *ρ*_1_ and *ρ*_2_ are the electron densities of heterogeneity domains (*φ*_1_ + *φ*_2_ = 1) in a two-phase system. A comparison of the values of invariant *Q*′ for the studied polymer systems (Table 1) shows that the relative level of the structural heterogeneity increases significantly during the transition from the IPEC and the IMC into the nanocomposites based on the IPEC and Ag^0^.

**Table 1:** Structural parameters and temperature transitions for the investigated polymer systems

Polymer system	*l*_*p*_, nm	*Q′*, rel. units	*T*_*g*_, °C	*T*_*f*_, °C	*ε*, % (*T* = 140 °C)
IPEC	35	10	63	207	23
IMC	43	24	57	199	18
IPEC–Ag^0^	15	39	65	212	10

An evaluation of the effective sizes of the heterogeneity regions existing in these polymer systems was performed through the method from [23, 24] via calculation of structural parameters, such as the range of heterogeneity (range of inhomogeneity), *l*_*p*_, which is directly related to the average diameters of heterogeneity regions, <*l*_1_> and <*l*_2_>, in the two-phase system:

$$ {l}_p={\varphi}_2<{l}_1>={\varphi}_1<{l}_2>. $$

After getting the result of the *l*_*p*_ calculation, the transition from the IPEC to the IMC was found to be accompanied with increasing of the effective size of region heterogeneity, while at transforming from the IMC into the PEC–Ag^0^ nanocomposites, effective size of heterogeneity decreased almost threefold (Table 1).

Alongside with the structural organization of the IPEC, the IMC, and the nanocomposites based on IPEC–Ag^0^, their thermomechanical behavior was studied.

**Fig. 4:**
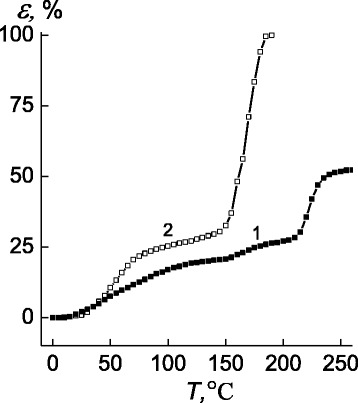
Thermomechanical curves of *1* pectin, and *2* P4VP. *ε* is relative value of penetration

Analysis of the pectin thermomechanical curve (see Fig. 4, curve 1) demonstrated that temperature transitions which are associated with the glass transition and melting of the pectin crystallites occur in the temperature ranges 20–110 and 155–230 °C, respectively. Also, the strong deformational change has been observed in the melting process of pectin’s crystalline phase [26]. As a contrast to anionic PE, the P4VP’s thermomechanical curve has the usual (typical) shape with glass transition interval between 25 and 80 °C and flow temperature from 150 to 180 °C (curve 2).

**Fig. 5:**
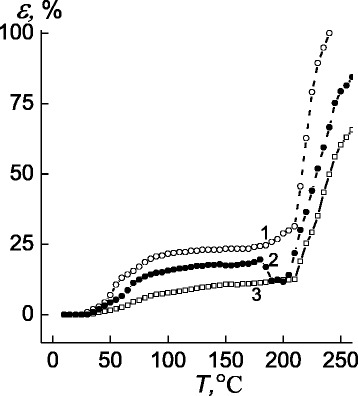
Thermomechanical curves of *1* the IPEC pectin–P4VP, *2* the IMC pectin–Ag^+^–P4VP, and *3* the IPEC–Ag^0^ nanocomposite

So, the shape of thermomechanical curve for IPEC (pectin–P4VP formed of equimolar quantities of anionic and cationic PE) is similar to P4VP’s one with glass transition temperature in the range between 30 and 85 °C and flow point from 180 to 240 °C (Fig. 5, curve 1).

Transferring from IPEC (pectin–P4VP) to the IMC (pectin–Ag^+^–P4VP), on the latter curve, a temperature shift at *T* = 200 °C appears, due to the AgNO_3_ melting in the bulk of IPEC (curve 2).

It is possible to conclude that in the temperature range 160–200 °C, the following processes take place: destructing of interpolyelectrolyte–metal complexes, transfering of AgNO_3_ salt from ionic state to the crystalline one, and, finally, its melting. So, changes occurred on the way from IPEC to IMC and to IPEC–Ag^0^ nanocomposite demonstrate the level of relative penetration in these systems tends to be decreasing (Fig. 5).

Basing on the data of polymeric objects depicted in Fig. 5, the average-interval temperature values of glass transition, fluidity temperature, and relative penetration (in the high-elasticity state at *T* = 140 °C) have been determined (Table 1).

## Conclusions

The investigations conducted involving wide-angle X-ray analysis revealed that transformation from a pectin–P4VP IPEC to a pectin–Ag^+^–P4VP IMC leads to the appearance in the X-ray profile a diffuse diffraction peak with 2*θ*_*m*_ ~11.0°, which characterizes the existence of interpolyelectrolyte–metal complexes.

Applying small-angle X-ray method, we confirmed that at transformation from IMC to IPEC–Ag^0^ structure of nanocomposite, the relative heterogeneity level is augmented, while effective size of the region heterogeneity goes down.

The chemical reduction of Ag^+^ ions in the IMC bulk by sodium borohydride leads to formation of the nanocomposite based on the “pectin–poly(4-vinylpyridine)” interpolyelectrolyte complex and Ag^0^ nanoparticles.

Nanocomposites IPEC–Ag^0^ are shown to possess a much higher values of glass transition temperatures and relative deformation compared to IMC.

## Abbreviations

IMC: Interpolyelectrolyte–metal complexes
IPEC: Interpolyelectrolyte complexes
P4VP: Poly(4-vinylpyridine)
PE: Polyelectrolytes
SAXS: Small-angle X-ray scattering
WAXS: Wide-angle X-ray scattering
